# Protein interactions: anything new?

**DOI:** 10.1042/EBC20220044

**Published:** 2022-12-16

**Authors:** Susana Barrera-Vilarmau, João M.C. Teixeira, Monika Fuxreiter

**Affiliations:** 1 Department of Biomedical Sciences, University of Padova, Italy; 2 Department of Physics and Astronomy, University of Padova, Italy

**Keywords:** energy landscape framework, fuzziness, higher-order assembly, liquid-liquid phase separation, protein-protein interactions

## Abstract

How do proteins interact in the cellular environment? Which interactions stabilize liquid–liquid phase separated condensates? Are the concepts, which have been developed for specific protein complexes also applicable to higher-order assemblies? Recent discoveries prompt for a universal framework for protein interactions, which can be applied across the scales of protein communities. Here, we discuss how our views on protein interactions have evolved from rigid structures to conformational ensembles of proteins and discuss the open problems, in particular related to biomolecular condensates. Protein interactions have evolved to follow changes in the cellular environment, which manifests in multiple modes of interactions between the same partners. Such cellular context-dependence requires multiplicity of binding modes (MBM) by sampling multiple minima of the interaction energy landscape. We demonstrate that the energy landscape framework of protein folding can be applied to explain this phenomenon, opening a perspective toward a physics-based, universal model for cellular protein behaviors.

## Introduction

Biological processes are achieved through different protein communities that range from binary complexes to cellular bodies. Specific rules governing the formation of these organizations also regulate the spatial and temporal characteristics of the underlying biological activities. How are these rules encoded in the amino acid sequence enabling protein function under a variety of cellular conditions? Recent discoveries highlight that, in addition to specific protein complexes, higher-order assemblies, ranging from ordered amyloids to dense liquid droplets, contribute to a wide range of biological activities [1]. In particular, there is an emerging interest in physiological and pathological roles of biomolecular condensates, generated by liquid–liquid phase separation [2–5]. Are the principles governing the formation of higher-order assemblies different from those driving specific protein assembly? Do we need new rules to describe liquid–liquid phase separation of biopolymers, or is it possible to apply existing models? How can we describe the delicate balance between stoichiometric and higher-order protein assemblies [6]? The aim of the present article is to discuss the principles of protein assembly across all scales of protein communities.

The article is organized as follows: First, we give a historical overview of protein interactions, introducing ordered and disordered binding modes. Second, we describe the phenomenon of context-dependent interactions. Third, we introduce the landscape model to describe interaction behaviors. Fourth, we propose the energy landscape framework as a universal model for protein folding and higher-order assembly.

## Interactions via ordered and disordered binding modes

**Figure 1:**
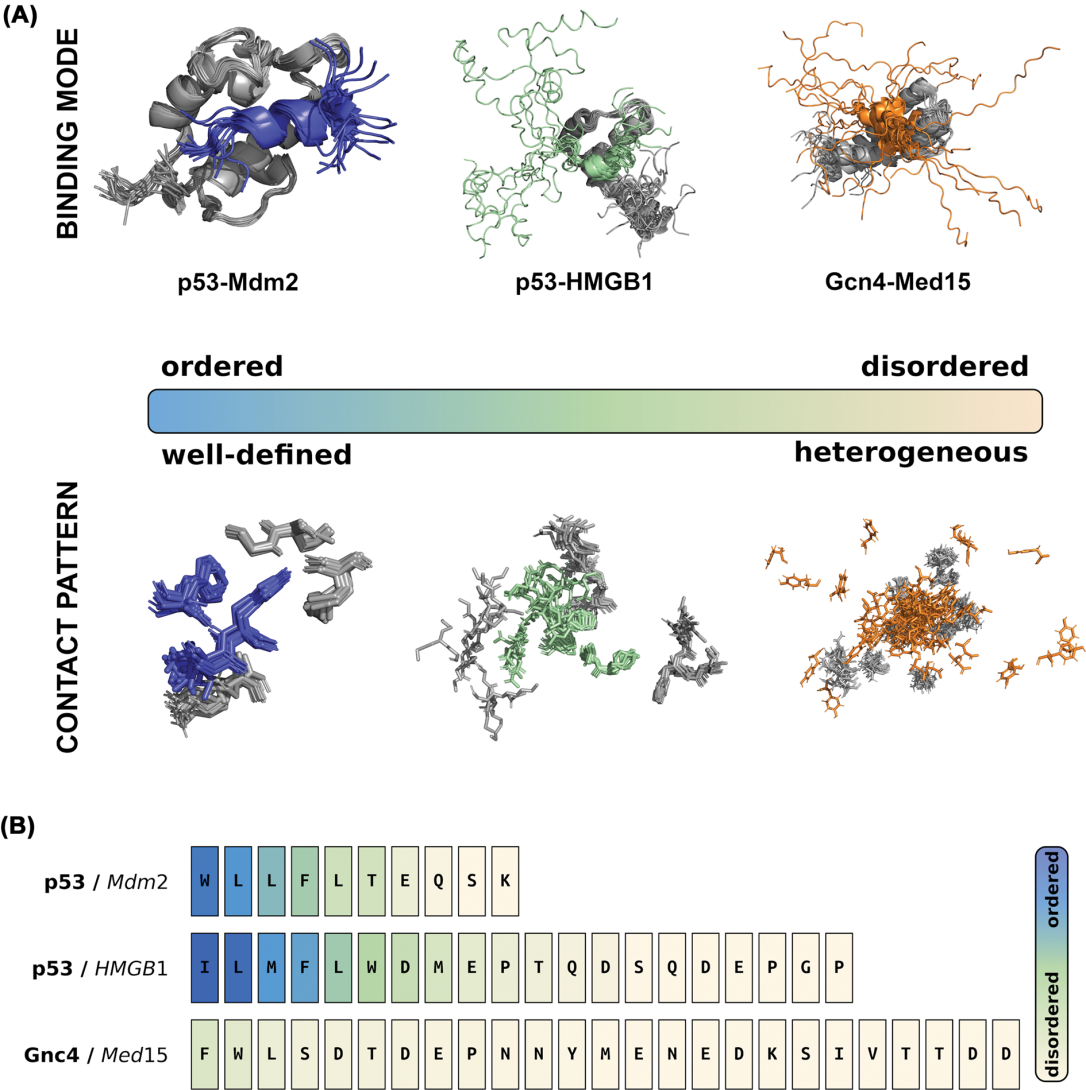
Protein interactions range from ordered to disordered binding modes (**A**) Structural order and contact patterns of the bound complex. In ordered binding modes, represented by the complex of p53 tumor suppressor (blue) with Mdm2 (gray, PDB: 2MWY, [12]), the binding interface is well-defined, the bound conformations exhibit limited mobility. Ordered binding modes are characterized by a well-defined contact pattern between specific residues. In disordered binding modes, represented by the complex formed between Gcn4 transcription factor (orange) and Med15 (gray, PDB: 2LPB [18]), the binding interface is conformationally heterogeneous as either or both partners are disordered in the bound complex. Disordered binding modes are generated by alternative contact patterns among the same set of residues. In context-dependent binding modes, represented by the complex formed between p53 (green) and HMGB1 (gray, PDB: 2LY4, [11]) the degrees of disorder are modulated by the cellular or experimental conditions. In context-dependent binding modes, some contacts are well-defined, whereas others are variable and depend on the conditions. The upper panels show the structure of the bound complex, while the lower panels display the residues, which are in contact in at least one model of the ensemble. (**B**) Heterogeneity of contact patterns**.** The variability of interactions can be quantified by the contact frequency, defined as the fraction of models in which the given residue is observed to form a contact (defined by https://getcontacts.github.io). The color scale ranges from more stable (blue) to more transient (light orange) interactions corresponding to highly or sparsely populated contacts. The interface residues of p53 exhibit more stable, well-defined contacts in complex with Mdm2, while more variable contacts with HMGB1. In contrast, Gcn4 forms contacts through any of its residues with a low frequency, leading to contact pattern variability and disordered binding modes.

The binding mode in stoichiometric protein complexes is usually classified based on the structural changes upon interactions. The binding mode is classified as ‘ordered’ if the resulted complex has a well-defined structure in the bound state, while ‘disordered’ if the complex samples an ensemble of conformations (Figure 1). In the present review, we classify the binding modes based on the contact pattern between the interacting residues [7,8]. The relationship between these two classifications may not be straightforward. Disordered protein regions can undergo templated folding upon binding and form well-defined structures [9]. This process usually results in a unique contact pattern between specific residues, in case the observed secondary structure element has a fixed position (Figure 1A). However, in case the secondary structure remains mobile (e.g. exhibits rigid body motions), we observe ambiguous contacts among a given set of residues. The transactivation domain of the p53 tumor-suppressor adopts an α-helical conformation upon binding to Mdm2 [10] as well as HMGB1 [11]. While the p53 helix formed upon interacting with Mdm2 ubiquitin ligase is relatively rigid (PDB: 1YCR [10], 2MWY [12]), the helix formed with HMGB1 remains mobile in the complex (PDB: 2LY4 [11]) (Figure 1A). In this manner, templated folding can result in a unique complex with a well-defined (unambiguous) contact pattern or a set of conformations with ambiguous contact patterns.

Alternatively, disordered proteins may remain disordered in their bound states forming conformationally heterogeneous complexes [13,14] with heterogeneous contact patterns [15] (Figure 1A). Disordered binding modes can also be achieved by partial unfolding of ordered proteins [16] or enhancing dynamics of disordered proteins [17]. This binding mode involves multiple (alternative) residues in physical interactions, which can be illustrated by the complex formed between Gcn4 and Med15 (PDB: 2LPB) [18], where the recognition element of the transcription factor exhibits considerable motions while attached to the shallow-binding cleft of the cofactor (Figure 1A). The disordered binding mode involves all residues of Gcn4 (Figure 1B), except F124 and W120, which serve as an anchor points for the rotation. Importantly, transcriptional activity is compromised by impeding the movement of the helix and the resulted interaction ambiguity [19].

These two scenarios are associated with distinct sequence features. High complexity sequence motifs can form a well-defined interaction pattern, while low complexity motifs form ambiguous interaction patterns [20] (Figure 1B). This can be quantified by the differences between the compositions of the interacting motifs and their flanking sequences, defined as the local sequence bias [20]. It has been demonstrated that strong sequence bias in the binding site leads to ordering and specific contacts, while a weak sequence bias decreases order and leads to ambiguity in contacts [20]. For example, repetitive sequence motifs or multiple binding sites decrease the local sequence bias and lead to disordered binding, for example, as observed in liquid–liquid phase-separated droplets [21].

Taken together, proteins may interact via a wide range of binding modes, from ordered binding mode with a well-defined contact pattern to disordered binding modes with heterogeneous contact patterns, which are encoded in the local complexity of the protein sequence.

## Evolutionary aspects of different binding modes

The experimentally observed binding characteristics reflect the binding mode of the interaction. The dissociation constant of disordered proteins with ordered binding modes strongly depends on the flanking sequences, which can modulate the affinity by orders of magnitude [22,23]. Flanking sequences may affect the stability of the binding element or perturb electrostatic interactions often leading to autoinhibitory effects [24]. Increased structural stability of binding elements may not improve biological activity. In case of p27^Kip1^ cell-cycle kinase inhibitor, for example, increased stability of the α-helix motif compromises the inhibitory function [25]. In addition, the flanking sequences may also modulate the mobility of the binding element to fine-tune the entropy of binding [26].

In disordered binding modes, the affinity linearly depends on the length and the number of binding motifs involved [27], indicating the lack of cooperativity [15]. For example, binding of Tau to microtubules depends on the number of repeats in the construct used for the experiment [28]. Gradual truncation of these sequences usually results in gradual decrease in activity [29]. In contrast, removal of the interacting element in ordered binding modes diminishes binding [30].

Why does Nature use different binding modes for interactions? Evolvability requires sequence flexibility to avoid accumulation of lethal variations [31]. Thus, certain processes, like regulatory processes (e.g. gene-expression, cell-cycle, carbohydrate metabolism), exploratory processes (e.g. T-cell receptor activity, cytoskeleton morphogenesis), compartmentation (e.g. during specification), or establishing coupling between processes (i.e. weak linkages) require moderate constraints on the underlying sequence elements. Therefore, to promote evolvability, these processes are associated with versatile, redundant sequence elements to reduce the interdependence of components and confer robustness and flexibility on these processes [31]. Owing to these reasons, there is also an evolutionary selection for low-complexity sequences [32], which will interact via disordered binding modes. Interactions of low-complexity protein sequences can then be opportunistically exploited for functions, for example, generating cellular bodies, termed as membraneless organelles [33].

Taken together, different biological processes require proteins with different local sequence complexities, resulting in different binding modes for interactions.

## Cellular context-dependence of protein interactions

The cell responds to millions of stimuli. Nevertheless, biological processes are achieved with high accuracy under a wide variety of cellular conditions. For example, the Ets-1 transcription factor functions under a variable control to regulate stem cell differentiation [29]. Its gene-expression activity depends on the Ca^2+^ concentration, which modulates the binding affinity of Ets-1 to DNA by orders of magnitude through multisite phosphorylation of Ets-1, which has an autoinhibitory effect via regulating the stability of the interaction element [34]. Thus, conditions in the cellular environment are reflected by Ca^2+^-induced multisite phosphorylation, which modulates the mode of interaction between the transcription factor and its cognate sequence [29].

**Figure 2:**
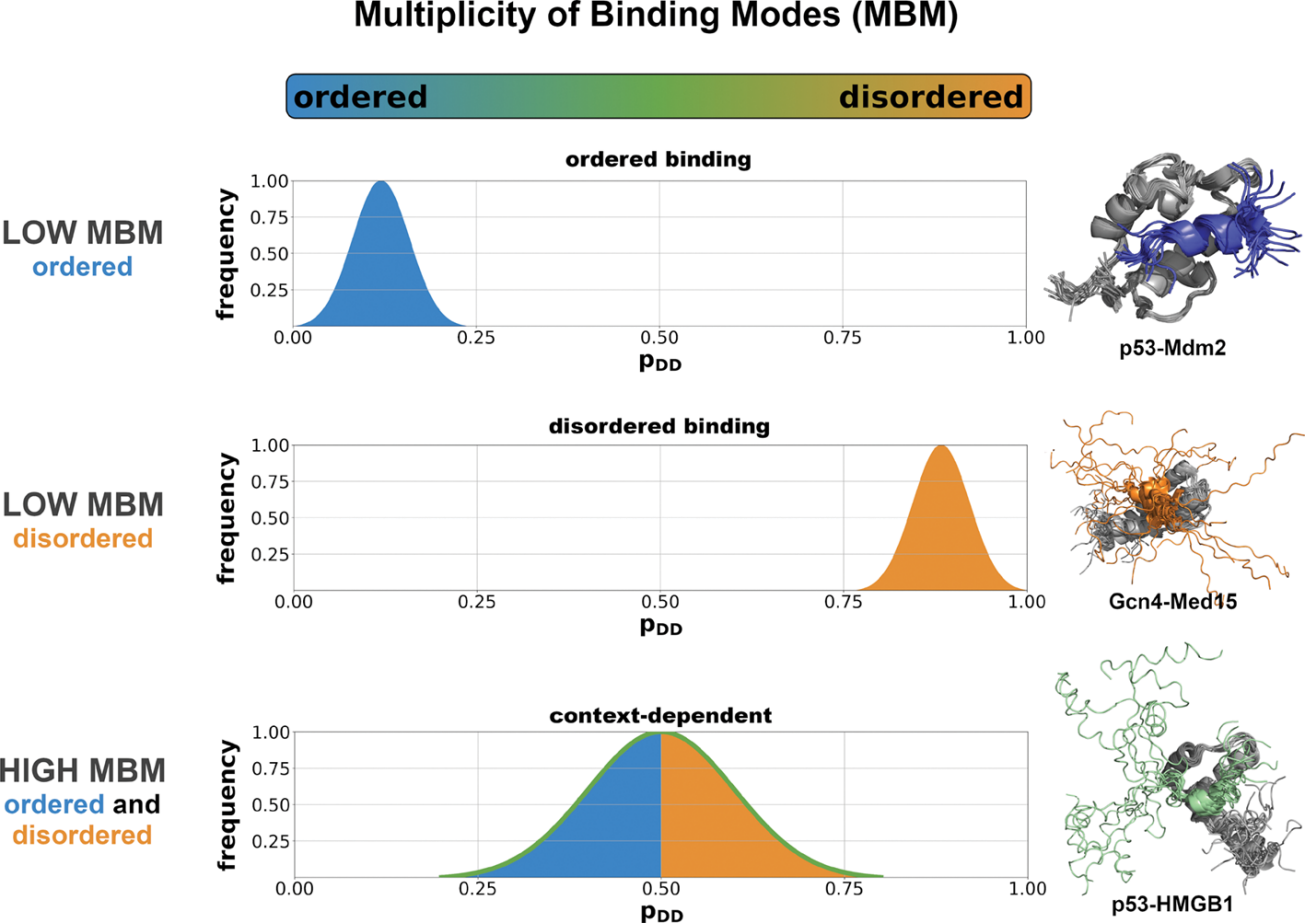
Protein interactions sample multiplicity of binding modes (MBM) Protein interactions with low MBM sample only one binding mode (unimodal), which can be ordered (blue, p53/Mdm2 complex [12]) or disordered (orange, Gcn4/Med15 complex [18]). Context-dependent protein interactions have high MBM, as they sample multiple binding modes (multimodal) in between ordered (blue) and disordered (orange) modes (p53/HMGB1 complex [11]). The distributions show the frequencies of different binding modes, which can be sampled under different cellular conditions or partners. These can be evaluated using the computational methods described in Ref. [38].

Overall, cellular conditions translate into changes in the binding modes of the interactions, with an impact on specificity, structural order or biological pathway [35]. In the insulin pathway, for example, the N-terminal region of glycogen synthase kinase 3 (GSK-3) binds to Axin in an ordered binding mode, whereas in the Wnt pathway the N-terminal region of GSK-3 remains disordered and interacts via a disordered binding mode [36]. This example illustrates that depending on the cellular conditions, proteins may exhibit a multiplicity of binding modes (MBM) [7,15] (Figure 2). Thus, similarly to their free states, proteins also sample a regime of their interaction space [37]. Variation in binding modes means that under certain conditions the bound state of proteins is confined to a single minimum, while under other conditions the bound conformations sample multiple minima (Figure 2). The interaction behavior is thus regulated by the cellular context, and the same protein region can establish a well-defined or alternative contact patterns depending on a variety of cellular factors, such as localization, ion or metabolite concentration, or post-translational modifications (Figure 2) [38,39]. To characterize the interaction behavior of proteins in the cellular environment, we define MBM as the likelihood of sampling multiple binding modes. The value of MBM is derived from the binding mode entropy [38] and is normalised to [0,1].

Liquid–liquid phase separation of proteins in particular depends on the cellular context [40]. Droplet formation of Tau, for example, is influenced by both the phosphorylation [41] as well as by the interaction partner EFhd2 [42]. Cellular localization and the protein quality control system also influences the formation and biophysical properties of condensates [43,44]. The importance of context-dependent interactions in regulation of higher-order assemblies [1], and in particular condensate assembly/disassembly is increasingly recognized [45]. Amyloid aggregation within liquid-like protein droplets is an example of a change in binding modes [46,47]. Protein droplets are associated with disordered binding modes, while amyloids are associated with ordered binding modes. Upon droplet maturation, the disordered binding mode is gradually converted into an ordered binding mode [48]. Indeed, sequence elements, with increased likelihood to change their binding modes, serve as aggregation hot spots [46].

**Figure 3:**
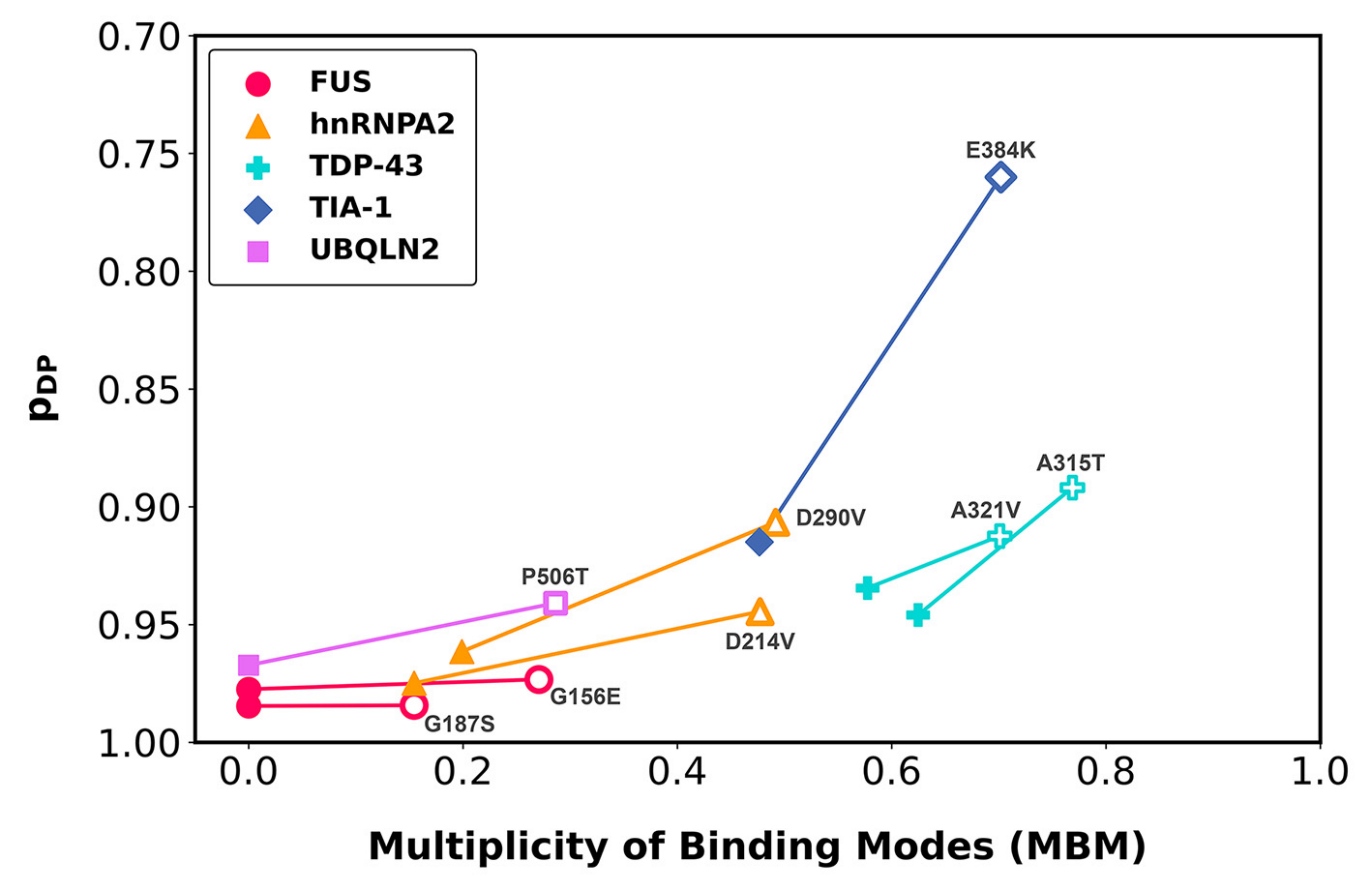
The droplet landscape indicates an impact of ALS-associated mutations on the multiplicity of binding modes (MBM) The droplet landscape represents the change in the cellular state as a function of MBM (x-axis) and droplet-promoting probability *p_DP_* (y-axis) in ALS-associated mutations (empty symbols) versus the wild-type sequence (filled symbols). On the droplet landscape, ALS-associated mutations of droplet-forming proteins, such as FUS G156E [49], G187S [50] (red circle); TDP-43 A321V [51], A315T [52] (cyan cross); TIA-1 E384K [53] (blue diamond); hnRNPA2 D214V [54], D290V [55,56] (orange triangle); UBQLN2 P506T [57] (purple square) are shown. As compared with the wild-type sequences (filled symbols), ALS mutations (open symbols) significantly impact MBM (shift to right), while exhibiting a negligible decrease in droplet-promoting probability (shift upward), which is proportional to the increase in ordered binding modes. Data are derived from Ref. [46]. The MBM values were derived from the binding mode entropy [38] and were normalised to [0,1].

To illustrate this point, we have analyzed mutations in droplet-forming proteins, FUS (G156E [49], G187S [50]), TDP-43 (A321V [51], A315T [52]), TAU (P301L [41]), TIA-1 (E384K [53]), hnRNPA2 (D214V [54], D290V [55,56]), UBQLN2 (P506T [57]), which are associated with amyotrophic lateral sclerosis (ALS). We have computed changes in the droplet-promoting probability (*p_DP_*), which is proportional to the probability of sampling disordered binding modes [21] as well as the change in multiplicity of binding modes (MBM) [46]. We found that mutations with diverse chemical nature (G→E, G→S, D→V, P→L, A→V, A→T, E→K, P→T) reduce the probability of droplet-formation to a small extent, significantly impact MBM, and expand the binding mode repertoire toward ordered binding modes. The droplet landscape represents the change in the cellular state as a function of MBM and droplet-promoting probability (Figure 3). ALS-mutants of droplet-forming proteins are shifted toward the right as compared with the wild-type sequences, exhibiting an increase in multiplicity of binding modes (Figure 3). In turn, most mutants exhibit only a negligible increase in the probability ordered binding modes. This indicates an that expansion of the binding mode repertoire, while gradually sampling more ordered binding modes towards solid-like aggregates.

Taken together, protein interactions follow changes in the cellular environment and context-dependence is linked with multiplicity of binding modes.

## The energy landscape framework as a universal physical model

Protein structures are usually interpreted within the energy landscape framework. The polypeptide chain aims to achieve an energy optimum during folding, which is often associated with the minimum of a funnel. Because of the many-body problem, not all interactions can be optimized simultaneously, leading to suboptimal contacts and energetic frustration [58]. It was demonstrated almost 50 years ago that even the most ordered proteins, such as myoglobin, can sample suboptimal alternative states; represented by the ruggedness of the energy landscape. In a biological sense, suboptimal states may represent functional variations and enhance adaptability [59]. Similarly, the energy landscape of a protein interaction may not be fully optimized and ruggedness can lead to functional variations [37].

**Figure 4:**
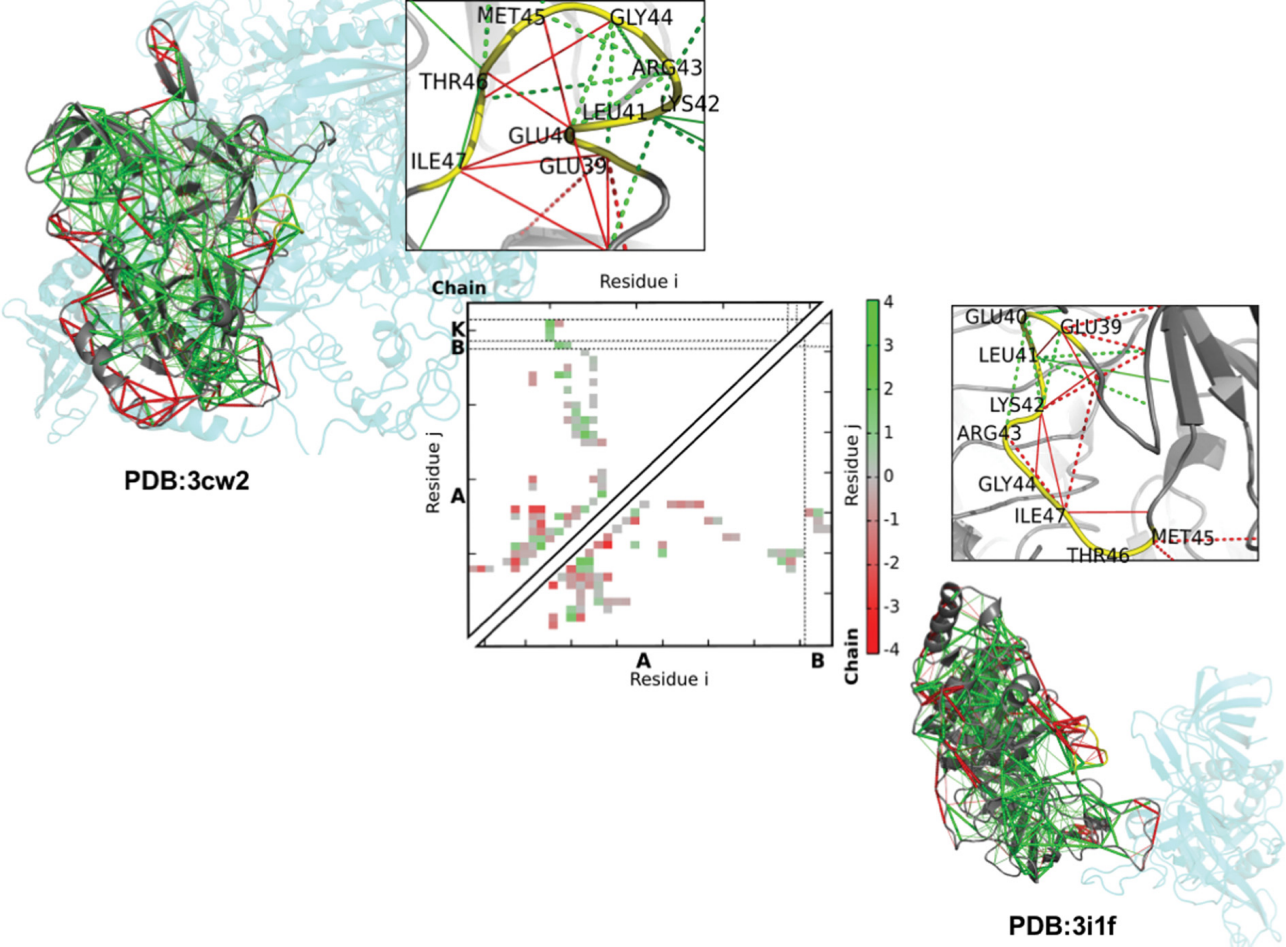
Energetic frustration in specific protein complexes Structures for translation initiation factor 2 (eIF2g) subunit gamma in complex with alpha and beta (PDB: 3CW2 [66], upper left) and in complex with a substrate-mimicking small molecule (PDB 3I1F; lower right). The disordered binding region, which undergoes templated folding, is shown with the yellow backbone on the insets. Contact patterns (3CW2 upper triangle; and 3I1F lower triangle) show the local frustration patterns of the protein, with the minimally frustrated interactions in green, the neutral shown in gray, and highly frustrated interactions in red. The contact patterns indicate that both complexes are energetically suboptimal, yet the pattern of frustrated contacts are different, enabling specific associations. This figure was adapted from Ref. [37], and is reproduced with permission. Copyright 2021, American Chemical Society.

Structures formed through templated folding of disordered proteins upon binding their partners comprise high density of suboptimal contacts [60]. This indicates that adopting a well-defined conformation may cause frustration both in the disordered protein as well as in the binding partner. It appears that frustration in folding and binding are likely related [37]. Thus, the interaction energy landscape will be rugged and the failure of simultaneously optimizing all intermolecular interactions leads to suboptimal, alternative-bound states. For example, binding-coupled folding of proteins with multiple interaction sites will generate high energetic frustration [60,61]. We observed that specific complexes of disordered proteins are energetically suboptimal, yet exhibit distinct frustration patterns in different complexes (Figure 4). This illustrates that specificity can be achieved without an energetic optimum [60].

The analogy between the folding and interaction energy landscapes [37] suggests that suboptimal states of the folding landscape can be stabilized by intermolecular interactions; for example, by higher-order organization of proteins. In particular, at high cellular concentrations, in the condensed states, the amyloid state, becomes thermodynamically favored [62]. Both condensed states, the liquid-like droplet state and the solid-like amyloid state, are stabilized by non-native interactions [21]. These non-native interactions generate ruggedness of the folding landscape to regulate the native function at low concentrations, while govern the formation of higher-order states at high cellular concentrations [6].

The energy landscape framework, for example, has been successfully applied to explain different mechanisms of amyloid aggregation [63]. Different Tau isoforms can form amorphous (K18) or prefibrillar (K19) oligomers, respectively, which cannot be readily converted into each other. Switching between the different pathways involving the distinct oligomer forms is thus coupled to structural reordering (‘backtracking’) [63]. In addition, frustrated contacts are affected by context-dependent phosphorylation, which favors the formation of large, amorphous oligomers in case of Tau K18 isoform, consistent with the experimental observations [41].

As the same physical principles drive organization of the polypeptide chain at different levels, the energy landscape framework is universal, from stoichiometric specific complexes to nonstoichiometric higher-order assemblies.

## Conclusions and outlook

Cellular organizations of proteins present a challenge to relate sequence and biological activity. Depending on the associated processes, protein sequences have evolved to high or low complexity. While structure–function relationships of high complexity sequences can be described by deterministic models, these cannot be readily applied to low-complexity sequences [8,64]. At low protein concentrations, specific interactions between high-complexity sequences dominate, while at high concentrations non-native contacts by low-complexity regions are favored [6]. Thus, at high protein concentrations such as in the cell, most proteins sample three fundamental states: the native state, the liquid-like droplet state, and the solid-like amyloid state [6]. Along these lines, the higher-order organization of proteins should be considered in developing structure-function relationships under cellular conditions.

Experimental results highlight the complexity of the protein interactions, in particular in the cellular environment, where assembly/disassembly is regulated by a wide variety of conditions. Therefore, proteins sample a wide range of binding modes, from ordered to disordered binding. Many proteins are also capable of interacting through a multiplicity of binding modes (MBM), resulting in variable behaviors according to the cellular context [35]. Proteins with high MBM can shift between ordered and disordered binding, such as in case of maturation of the droplet state to the amyloid state [46,47]. Different binding modes can be associated with distinct pathways, and regions with high MBM may switch between these activities [36]. In a similar manner, shifting binding modes by post-translational modifications can induce assembly/disassembly of biomolecular condensates formed by liquid-liquid phase separation [36].

It is challenging to decipher how the interaction behavior, quantified by MBM, is encoded in the protein sequence. In contrast to the traditional process of identifying specific binding motifs, one needs to investigate the local sequence bias of the potential interaction sites. The local sequence bias determines the ordered or disordered binding modes of interactions. Because of context-dependence, the interaction motifs will change with the cellular conditions. Thus, if the local sequence bias can be modulated considerably by the minor alterations in the flanking regions, for example, by post-translational modifications or by masking a few interaction sites, the binding element will be prone to sample high MBM. In contrast, if the local sequence bias is robust to alterations in the flanking regions, the interaction will be unimodal with low MBM. These principles are represented by binding [38] and droplet landscapes [46], which reflect the interaction characteristics in the native and droplet states and can be computed efficiently from the protein sequence.

The common origin of interaction characteristics in higher-order assemblies, for example, liquid–liquid phase-separated droplets and stoichiometric protein complexes, will facilitate deciphering the sequence codes for physiological functions and aberrant states. Identifying non-native interactions, based on energetic considerations, is more straightforward than identifying universal-binding motifs applicable to most proteins. Analysis of heterogeneous conformational ensembles can be helpful in this endeavor [15,65].

In summary, protein interaction complexity in the cellular environment is manifested in the assembly through multiplicity of binding modes. The physical origin of MBM is energetic frustration, and thus can be described within the energy landscape framework similarly to protein folding. Recognizing the common origin of protein folding and higher-order assembly will facilitate the development of physics-based, quantitative models for biomolecular condensates.

## Summary

- Proteins interact via a wide range of binding modes from ordered to disordered binding, from well-defined to ambiguous contact patterns.
- Protein interactions sample a multiplicity of binding modes (MBM) depending on the cellular context.
- MBM is critical for aggregation of liquid–liquid phase-separated droplets to solid-like amyloid fibers.
- The energy landscape framework is a universal, physics-based framework linking folding to assembly across all scales of protein communities.

## Abbreviations

ALS: amyotrophic lateral sclerosis
GSK-3: glycogen synthase kinase 3
MBM: multiplicity of binding modes
PDB: Protein Data Bank
*p*_DP_: droplet-promoting probability
